# Comparative Efficacy of BTK Inhibitors in Treatment‐Naïve and Relapsed/Refractory Mantle Cell Lymphoma: A Systematic Review and Meta‐Analysis

**DOI:** 10.1111/jcmm.71340

**Published:** 2026-09-02

**Authors:** Fangfei Xu, Xiuping Zou, Yulin Yang, Kuangguo Zhou, Wei Huang

**Affiliations:** ^1^ Department of Hematology Tongji Hospital, Tongji Medical College, Huazhong University of Science and Technology Wuhan Hubei China; ^2^ Immunotherapy Research Center for Hematologic Diseases of Hubei Province Wuhan Hubei China; ^3^ Suizhou Hospital Hubei University of Medicine Suizhou Hubei China

**Keywords:** Bruton tyrosine kinase inhibitors (BTKis), CAR‐T cell therapy, complete response rate (CR), mantle cell lymphoma (MCL), systematic review and meta‐analysis

## Abstract

Bruton tyrosine kinase inhibitors (BTKis) have been employed in the treatment of mantle cell lymphoma (MCL). However, direct comparisons of ibrutinib, zanubrutinib, and acalabrutinib across treatment‐naïve (TN) and relapsed/refractory (R/R) MCL remain limited. This meta‐analysis was intended to evaluate their efficacy, addressing critical gaps in clinical decision‐making. We systematically searched PubMed, Embase, and Cochrane up to January 2025 for studies (RCT/single‐arm) assessing the efficacy of BTKis in MCL patients. Among 70 studies, the pooled CR rate in the TN group was higher than that in the R/R group (76.5% vs. 43.2%). Among TN patients, the CR rate of the regimen incorporating zanubrutinib (95.2% [95% CI 0.893, 1.000]) was significantly higher than that of the regimens containing acalabrutinib or ibrutinib (*p* = 0.0042). In the R/R group, the BTKi + anti‐CD20 monoclonal antibody + small‐molecular therapy group presented a better CR rate (68.3% [95% CI 0.546, 0.820]; *p* < 0.0001). When comparing the monotherapy efficacy of three BTKis in R/R MCL, the results indicated that acalabrutinib exhibited a higher CR rate (43.2% [95% CI 0.339, 0.525]) than zanubrutinib or ibrutinib. In addition, zanubrutinib‐based therapy exhibited a lower pooled rate of haematological toxicities compared to the other two BTKi therapies. This work resolved critical uncertainties in BTKi selection for MCL, demonstrating acalabrutinib's and zanubrutinib's first‐line potential, leading to a meaningful improvement in response rate and a manageable safety profile. Chemotherapy‐free regimen can partially overcome the traditionally unfavourable prognosis associated with R/R MCL. These results provide a roadmap for optimizing MCL therapy. Chemotherapy‐free regimens for MCL based on BTKis warrant further validation in RCTs. These findings may advocate for updated guidelines prioritizing zanubrutinib and acalabrutinib in clinical practice.

## 1 Introduction

Mantle cell lymphoma (MCL), which accounted for 5%–7% of all lymphoma cases, posed formidable treatment challenges [1]. MCL was characterized by a poor prognosis due to high relapse rates and resistance to conventional therapies such as chemotherapy. For younger patients who were eligible for autologous stem cell transplantation (ASCT), ASCT could extend the median progression‐free survival (PFS) to 7–10 years [2, 3, 4]. Not all patients were suitable for ASCT, and unfortunately, most of these patients eventually suffered relapse. Recent retrospective studies indicated that the 5‐year overall survival (OS) rate for patients who were not candidates for ASCT was below 65% [5, 6]. Molecular drivers such as the t(11;14) translocation (leading to Cyclin D1 overexpression) and TP53 mutations further exacerbated disease aggressiveness, particularly in high‐risk subgroups [7]. Treatment of relapsed/refractory (R/R) MCL is particularly difficult because rituximab has limited efficacy [8].

The advent of Bruton tyrosine kinase inhibitors (BTKis) has revolutionized MCL management. Ibrutinib, a first‐generation BTKi, improved PFS in R/R MCL [9]. Second‐generation BTKis, zanubrutinib and acalabrutinib, were developed to enhance their selectivity and safety [10, 11]. Notably, acalabrutinib recently became a preferred first‐line treatment option (combined with bendamustine/rituximab) for MCL in the NCCN 2025 guideline, based on the ECHO trial showing superior complete response (CR) and PFS (CR: 88.9%; median PFS: 28.6 months) [12]. Zanubrutinib, while approved for frontline use, became a first‐line treatment option only for TP53‐mutant MCL owing to unprecedented CR rates (88% in treatment‐naïve TP53‐mutant cohorts) in Phase II trials [13].

Despite these advancements, there remain crucial gaps in clinical decision‐making. Firstly, there are uncertainties regarding stage‐specific efficacy. Current guidelines recommend acalabrutinib as the preferred first‐line treatment option for MCL, restrict zanubrutinib to first‐line use in TP53‐mutated MCL, and limit the use of ibrutinib to patients with R/R disease, primarily due to heterogeneous trial designs [12, 13, 14, 15]. Secondly, evidence gaps in combination strategies also exist. In addition to BTKis and traditional chemotherapy, molecularly targeted therapies such as rituximab, lenalidomide, venetoclax, bortezomib, as well as chimeric antigen receptor (CAR) T‐cell therapy have also been recommended in the NCCN 2025 guidelines. Because of the heterogeneity in the design of clinical studies, the lack of comparability among BTKi‐containing combination regimens led to confusion in clinical decision‐making.

The objective of this systematic review and meta‐analysis was to provide the first comprehensive evidence to refine clinical guidelines, optimize therapeutic sequencing, and address unmet needs in MCL management.

## 2 Methods

### 2.1 Search Strategy and Selection Criteria

Systematic searches of PubMed, Cochrane and Embase were conducted before January 31, 2025, utilizing relevant MeSH terms and keywords. The detailed search strategies were presented in Table S1–S3.

Included studies were as follows: (1) Studies were clinical trials (randomized controlled trials or single‐arm clinical trials); (2) Patients were diagnosed with mantle cell lymphoma; (3) Patients received BTKis as monotherapy or in combination with other therapies; (4) Studies reported at least one of the following clinical outcomes: CR, objective response rate (ORR); (5) Studies were published in English. Studies not meeting these criteria were excluded.

The meta‐analysis was reported in accordance with the Preferred Reporting Items for Systematic Reviews and Meta‐Analyses (PRISMA) guidelines. This systematic review has been registered with PROSPERO (CRD420251034251).

### 2.2 Data Extraction

Extracted details included the study details (author, publication year), patients' characteristics (number of patients, age), outcomes (CR rate, ORR, median PFS and median overall survival), and the number of adverse effects (AEs) including haematological AEs (neutropenia, thrombocytopenia, anaemia) and non‐haematological AEs (hepatotoxicity, cardiac events, haemorrhage, infection). Quality assessments were conducted independently by two reviewers using the Cochrane risk of bias 2 tool for RCTs and the MINORS assessment tool for single‐arm cohort studies. Any disagreements were resolved through discussion.

### 2.3 Statistical Analysis

The statistical analysis was conducted using R (version 4.2.2). Heterogeneity between studies was assessed using the I^2^ statistic. Studies with an *I* ^2^ value exceeding 50% were considered to have significant heterogeneity, and random‐effect models were employed. A fixed‐effect model was utilized when heterogeneity was low. Sensitivity analysis was performed by excluding each study one by one from the pooled results with high heterogeneity. Subgroup analyses were performed based on the type of BTKi and the type of intervention. Publication bias was evaluated using funnel plots, Egger's test and Begg's test. Two‐sided *p* < 0.05 was considered statistically significant for all statistical analyses.

## 3 Results

### 3.1 Study Selection

**FIGURE 1:**
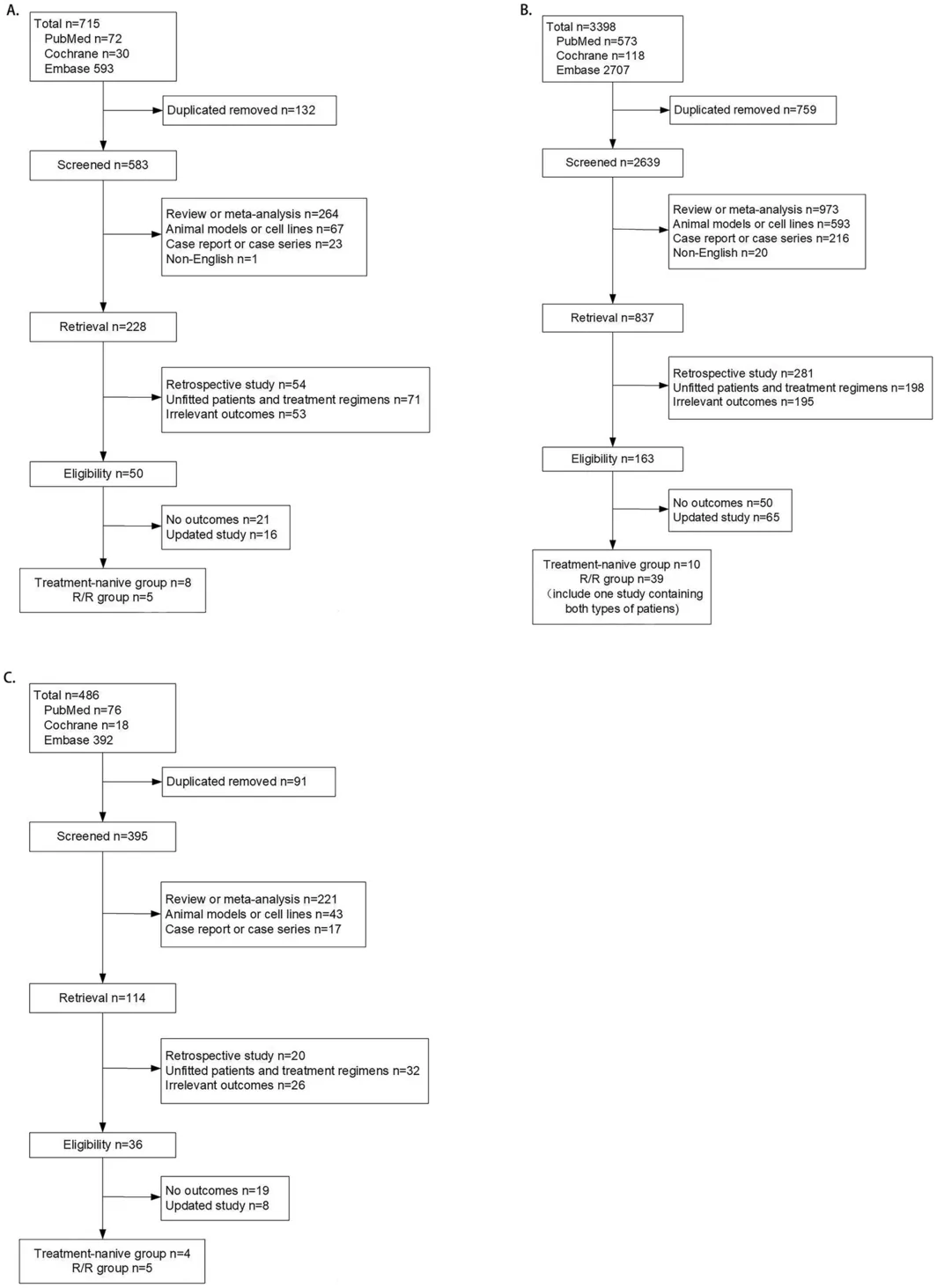
Flow diagram for study selection. (A) Acalabrutinib; (B) Ibrutinib; (C) Zanubrutinib. R/R, relapsed/refractory.

A comprehensive systematic search identified a total of 715 studies on acalabrutinib, 3398 studies on ibrutinib, and 486 studies on zanubrutinib. After the automatic removal of duplicates, 3635 independent studies remained. Two independent reviewers screened the titles and abstracts to exclude irrelevant articles. Any disagreements were resolved through discussion. Finally, 70 studies were included in this meta‐analysis (Figure 1) [12, 13, 15, 16, 17, 18, 19, 20, 21, 22, 23, 24, 25, 26, 27, 28, 29, 30, 31, 32, 33, 34, 35, 36, 37, 38, 39, 40, 41, 42, 43, 44, 45, 46, 47, 48, 49, 50, 51, 52, 53, 54, 55, 56, 57, 58, 59, 60, 61, 62, 63, 64, 65, 66, 67, 68, 69, 70, 71, 72, 73, 74, 75, 76, 77, 78, 79, 80, 81, 82]. The final analysis included 4 RCTs, 3 retro‐prospective observational studies, and 63 single‐arm cohort studies (Table 1).

**TABLE 1:** Overview of included studies.

Study	Design	Treatment	Sample size	Median age (range)	CR (%)	OR (%)
*Treatment‐naïve patients*						
Ruan et al. (2022) [16]	Phase 2, single‐arm	Acalabrutinib and lenalidomide during induction therapy; Acalabrutinib and rituximab during maintenance therapy	24	64 (35–77)	90.5	100
Wang et al. (2024) [17]	Phase 1b, single‐arm	Acalabrutinib, rituximab and venetoclax	21	66 (51–85)	74	100
Jain et al. (2023) [18]	Phase 2, single‐arm	Acalabrutinib and rituximab	50	69 (65–81)	90	94
Danilov et al. (2022) [19]	Phase 2, single‐arm	Acalabrutinib, umbralisib and ublituximab	12	70 (55–79)	100	100
Guy et al. (2020) [20]	Phase 2, single‐arm	Acalabrutinib+BR for cycles 1–3; Acalabrutinib+CR for cycles 4–6	12	57 (52–66)	75	83
Patel et al. (2023) [21]	Phase 2, single‐arm	Acalabrutinib+BR for cycles 1–3; Acalabrutinib+CR for cycles 4–6	13	61 (38–65)	69	85
Villa et al. (2023) [22]	Phase 2, single‐arm	Acalabrutinib and R‐CHOP	43	62 (38–69)	91	100
Hawkes et al. (2023) [23]	Phase 2, single‐arm	Acalabrutinib and rituximab for cycles 1–2; R‐DHAOx for cycles 3–6	44	59 (54–64)	88	95
Gaulin et al. (2023) [24]	Phase 2, single‐arm	Ibrutinib	20	60.5 (38–79)	NA	94.7
Tivey et al. (2024) [25]	Retro‐prosepctive observatory, single‐arm	Ibrutinib and rituximab	149	75 (41–94)	20.2	71.2
Gine et al. (2022) [26]	Phase 2, single‐arm	Ibrutinib and rituximab	50	65 (40–85)	80	84
Jain et al. (2022) [27]	Phase 2, single‐arm	Ibrutinib and rituximab	50	71 (69–76)	71	96
Le Gouill et al. (2021) [28]	Phase 1/2, single‐arm	Ibrutinib, obinutuzumab and venetoclax	15	65 (51–77)	86	93
Wang et al. (2022) [29]	Phase 2, single‐arm	Ibrutinib and rituximab	131	56 (49–60)	87	98
Tivey et al. (2022) [30]	Phase 3, single‐arm	Ibrutinib and rituximab	66	71 (41–89)	16.7	74.4
Wang et al. (2021) [31]	Phase 2, single‐arm	Ibrutinib, rituximab and venetoclax	50	57 (35–65)	92	96
Wang et al. (2022) [32]	RCT	Treatment group: Ibrutinib+BR; Control group: BR	Treatment group: 261	71 (65–86)	65.5	89.7
Dreyling et al. (2024) [33]	RCT	Group A + I: Ibrutinib, six alternating cycles of R‐CHOP and either R‐DHAP or R‐DHAOx, and ASCT; Group I: Ibrutinib, six alternating cycles of R‐CHOP and either R‐DHAP or R‐DHAOx	Group A + I: 292; Group I: 290	Group A + I: 57 (52–61); Group I: 57.5 (52–61)	45	98
Kumar et al. (2025) [13]	Phase 2, single‐arm	Zanubrutinib, obinutuzumab and venetoclax	25	68 (29–82)	88	96
Cai et al. (2024) [34]	Phase 2, single‐arm	Zanubrutinib and rituximab	42	57 (51–64)	91.9	NA
Qu et al. (2023) [35]	Phase 2, single‐arm	Zanubrutinib and rituximab	26	62	100	100
Yan et al. (2023) [36]	Phase 2, single‐arm	Zanubrutinib+*R*‐CHOP, and R‐DHAOx alternately	7	56.3	100	100
*Relapsed/refractory patients*						
Le Gouill et al. (2024) [37]	Phase 2, single‐arm	Acalabrutinib	124	68	47.6	81.5
Song et al. (2024) [38]	Phase 1/2, single‐arm	Acalabrutinib	34	63 (36–75)	35.3	82.4
Izutsu et al. (2021) [39]	Phase 1, single‐arm	Acalabrutinib	13	74 (55–82)	38	62
Kim et al. (2023) [40]	Phase 1/2, single‐arm	Acalabrutinib, obinutuzumab and venetoclax	9	67 (63–79)	67	78
Phillips et al. (2025) [12]	Phase 1b, single‐arm	Acalabrutinib and BR	20	65.0 (47–82)	70	85
Maruyama et al. (2019) [41]	Phase 2, single‐arm	Ibrutinib	16	72 (55–83)	31.3	93.8
Dartigeas et al. (2022) [42]	Retro‐prospective observatory, single‐arm	Ibrutinib	59	73 (49–88)	41.1	78.4
Rule et al. (2018) [43]	RCT	Ibrutinib vs. Temsirolimus	139	67	23	77
Wang et al. (2015) [44]	Phase 2, single‐arm	Ibrutinib	111	68 (40–84)	23	67
Vergote et al. (2022) [45]	Retro‐prospective observatory, single‐arm	Ibrutinib	76	74 (47–88)	39.5	93.4
Wang et al. (2014) [46]	Phase 2, single‐arm	Ibrutinib	120	67.5 (35–85)	20.9	62.7
Furtado et al. (2012) [47]	Phase 2, single‐arm	Ibrutinib	10	68	NA	62
Fowler et al. (2010) [48]	Phase 1, single‐arm	Ibrutinib	4	NA	0	50
Tucker et al. (2016) [49]	Phase 2, single‐arm	Ibrutinib	61	67 (48–90)	28	80
Tobinai et al. (2016) [50]	Phase 1, single‐arm	Ibrutinib	2	NA	0	50
Novak et al. (2023) [51]	Phase 1/2, single‐arm	Ibrutinib and bortezomib	55	71 (47–90)	16.4	81.8
Gurumurthi et al. (2024) [52]	Phase 1, single‐arm	Ibrutinib and abexinostat	8	NA	43	86
Lee et al. (2020) [53]	Phase 1, single‐arm	Ibrutinib and carfilzomib	8	68 (55–83)	0	50
Cohen et al. (2022) [54]	Phase 1/2, single‐arm	Ibrutinib and ixazomib	35	70 (53–84)	42.9	77.1
Christian et al. (2014) [55]	Phase 1, single‐arm	Ibrutinib and lenalidomide	2	NA	0	50
Wang et al. (2023) [56]	RCT	Treatment group: Ibrutinib and venetoclax; Control group: Ibrutinib and placebo	267	68	43.1	77.9
Portell et al. (2022) [57]	Phase 1, single‐arm	Ibrutinib and venetoclax	35	63 (49–82)	42.4	82.3
Jain et al. (2018) [58]	Phase 2, single‐arm	Ibrutinib and rituximab	50	68 (45–85)	58	88
Le Gouill et al. (2021) [28]	Phase 1/2, two cohorts	Cohort A: Ibrutinib and obinutuzumab; Cohort B: Ibrutinib, obinutuzumab and venetoclax	Cohort A: 9; Cohort B: 24	Cohort A: 64 (58–82); Cohort B: 66 (45–76)	Cohort A: 78； Cohort B: 67	Cohort A: 89; Cohort B: 71
Torka et al. (2023) [59]	Phase 1, single‐arm	Ibrutinib and pevonedistat	8	NA	50	100
Qualls et al. (2022) [60]	Phase 1, single‐arm	Ibrutinib and copanlisib	8	67	50	87.5
Goto et al. (2024) [61]	Phase 2, single‐arm	Ibrutinib and venetoclax	13	71 (59–81)	83	83
Stewart et al. (2022) [62]	Phase 1/1b, single‐arm	Ibrutinib and buparlisib	18	71 (50–84)	76	94
Kim et al. (2023) [63]	Phase 2, single‐arm	Ibrutinib and obinutuzumab	10	72 (55–81)	55.6	89
Stephens et al. (2022) [64]	Phase 1, single‐arm	Ibrutinib and selinexor	3	70 (66–74)	0	33
Handunnetti et al. (2024) [65]	Phase 2, single‐arm	Ibrutinib and venetoclax	23	68 (47–81)	73.9	78.3
Davids et al. (2019) [66]	Phase 1, single‐arm	Ibrutinib and umbralisib	21	68 (50–83)	19	67
Janakiram et al. (2021) [67]	Phase 1/2, single‐arm	Ibrutinib and Lonca	7	NA	NA	85.7
Martin et al. (2019) [68]	Phase 1, single‐arm	Ibrutinib and palbociclib	27	65 (42–81)	37	67
Lee et al. (2022) [69]	Phase 1/2, single‐arm	Ibrutinib and zilovertamab	33	NA	40.7	85.2
Lee et al. (2021) [70]	Phase 1/2, single‐arm	Ibrutinib and cirmtuzumab	20	NA	35	80
Sancho et al. (2024) [71]	Phase 1, three cohorts	Group A: Rituximab and parsaclisib; Group B: Rituximab, parsaclisib and bendamustine; Group C: Ibrutinib and parsaclisib	2	NA	50	100
Davids et al. (2017) [72]	Phase 1/1b, single‐arm	Ibrutinib and TGR‐1202 (umbralisib)	15	NA	7.7	85
Kolibaba et al. (2015) [73]	Phase 2, single‐arm	Ibrutinib and ublituximab	15	71 (55–80)	33	87
Jerkeman et al. (2018) [74]	Phase 2, single‐arm	Ibrutinib, rituximab and lenalidomide	50	69 (45–85)	56	76
Ip et al. (2023) [75]	Phase 1b, single‐arm	Ibrutinib, rituximab and lenalidomide	25	61 (39–79)	83	88
Grieve et al. (2024) [76]	Phase 1, single‐arm	Ibrutinib, rituximab, bendamustine and venetoclax	10	68 (60–81)	80	80
Bonnet et al. (2017) [77]	Phase 1b, single‐arm	Ibrutinib and R‐DHAP/R‐DHAOx	25	NA	50	87
Minson et al. (2024) [78]	Phase 1/2, single‐arm	Ibrutinib and CAR‐T	20	66 (41–74)	80	80
Song et al. (2022) [82]	Phase 2, single‐arm	Zanubrutinib	86	61 (34–75)	77.9	83.7
Tam et al. (2021) [15]	Phase 1/2, single‐arm	Zanubrutinib	32	70.5 (42–86)	25	84.4
Ishikawa et al. (2022) [79]	Phase 1/2, single‐arm	Zanubrutinib	11	NA	9.1	81.8
Soumerai et al. (2024) [80]	Phase 1b, single‐arm	Zanubrutinib and zandelisib	19	65 (52–78)	47	73.7
Stilgenbauer et al. (2024) [81]	Phase 1, single‐arm	Zanubrutinib and sonoro	35	NA	67	85

Abbreviations: ASCT, autologous stem‐cell transplantation; BR, bendamustine and rituximab; CAR‐T, chimeric antigen receptor T‐cell immunotherapy; CR, cytarabine and rituximab; R‐CHOP, rituximab, cyclophosphamide, doxorubicin, vincristine and prednisone; R‐DHAOx, rituximab, dexamethasone, cytarabine, oxaliplatin; R‐DHAP, rituximab, dexamethasone, cytarabine and cisplatin.

### 3.2 Study Characteristics

A total of 70 studies encompassed 1641 TN patients and 1791 R/R patients. The median age ranged from 56 to 75 years in the TN group and from 61 to 74 years in the R/R group (Table 1).

The combined treatment regimen encompassed traditional chemotherapy, anti‐CD20 monoclonal antibody therapy, small‐molecular therapy, CAR‐T, and other therapies. The traditional chemotherapies included CHOP (cyclophosphamide, doxorubicin, vincristine and prednisone), DHAOx (dexamethasone, cytarabine, oxaliplatin), DHAP (dexamethasone, cytarabine and cisplatin), bendamustine, cytarabine. The small‐molecular therapies included proteasome inhibitors (bortezomib, carfilzomib, ixazomib), venetoclax and lenalidomide, which were recommended in the National Comprehensive Cancer Network (NCCN) guideline (version 2025). The CAR‐T product used in the study was tisagenlecleucel. Other therapies, which were not recommended in the NCCN guideline (version 2025), included other molecularly targeted drugs such as abexinostat, zilovertamab, cirmtuzumab, sonrotoclax, umbralisib (TGR‐1202), copanlisib, buparlisib, parsaclisib, zandelisib, pevonedistat, selinexor, palbociclib, and loncastuximab tesirine.

### 3.3 Risk of Bias in Studies

Tables S4 and S5 presented the results of risk‐of‐bias assessments. For the single‐arm trials, the scores for “unbiased assessment of the study endpoint” were notably low, indicating a scarcity of blinded evaluations by the Independent Review Committee (IRC). However, the overall risk of bias of most studies was low. Regarding the four RCTs, Dreyling et al. [33] and Wang et al. [29] were assessed as being at low risk of bias, while Wang et al. [56] and Rule et al. [43]– raised some concerns.

### 3.4 The Efficacy of BTKis for MCL Patients

**FIGURE 2:**
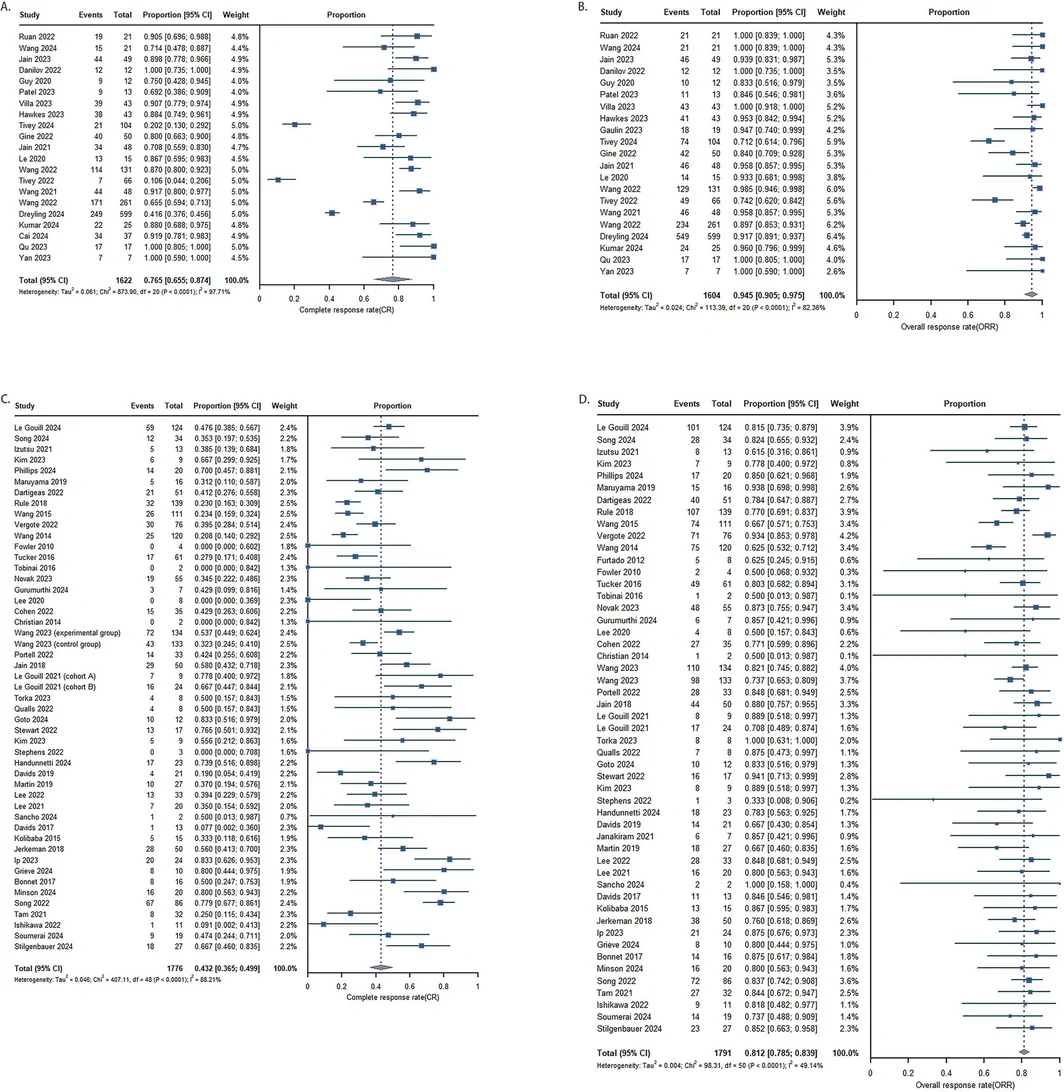
The forest plot for the effect of BTKis for MCL patients. (A) CR rate for treatment‐naïve patients. (B) ORR for treatment‐naïve patients. (C) CR rate for R/R patients. (D) ORR for R/R patients. BTKis, bruton tyrosine kinase inhibitors; CR, complete remission; ORR, objective response rate; R/R, relapsed/refractory.

21 studies in the TN group and 47 studies in the R/R group reported the CR rate, and 21 studies in the TN group and 49 studies in the R/R group reported the ORR. Although some studies have reported on PFS and overall survival (OS), the median PFS and OS were not reached, making it impossible to conduct a meta‐analysis. The pooled estimate for CR in TN and R/R MCL patients treated with BTKi therapy was 76.5% [95% CI 0.655, 0.874] and 43.2% [95% CI 0.365, 0.499], respectively, with high heterogeneity (*I* ^2^ = 97.71% in the TN group, *I* ^2^ = 88.21% in the R/R group; Figure 2A,C). The combined ORR of BTKi therapy was 94.5% [95% CI 0.905, 0.975] and 81.2% [95% CI 0.785, 0.839] in the TN group and the R/R group, respectively (Figure 2B,D).

### 3.5 Subgroup Analysis: Response Rates Per Type of BTKis

**FIGURE 3:**
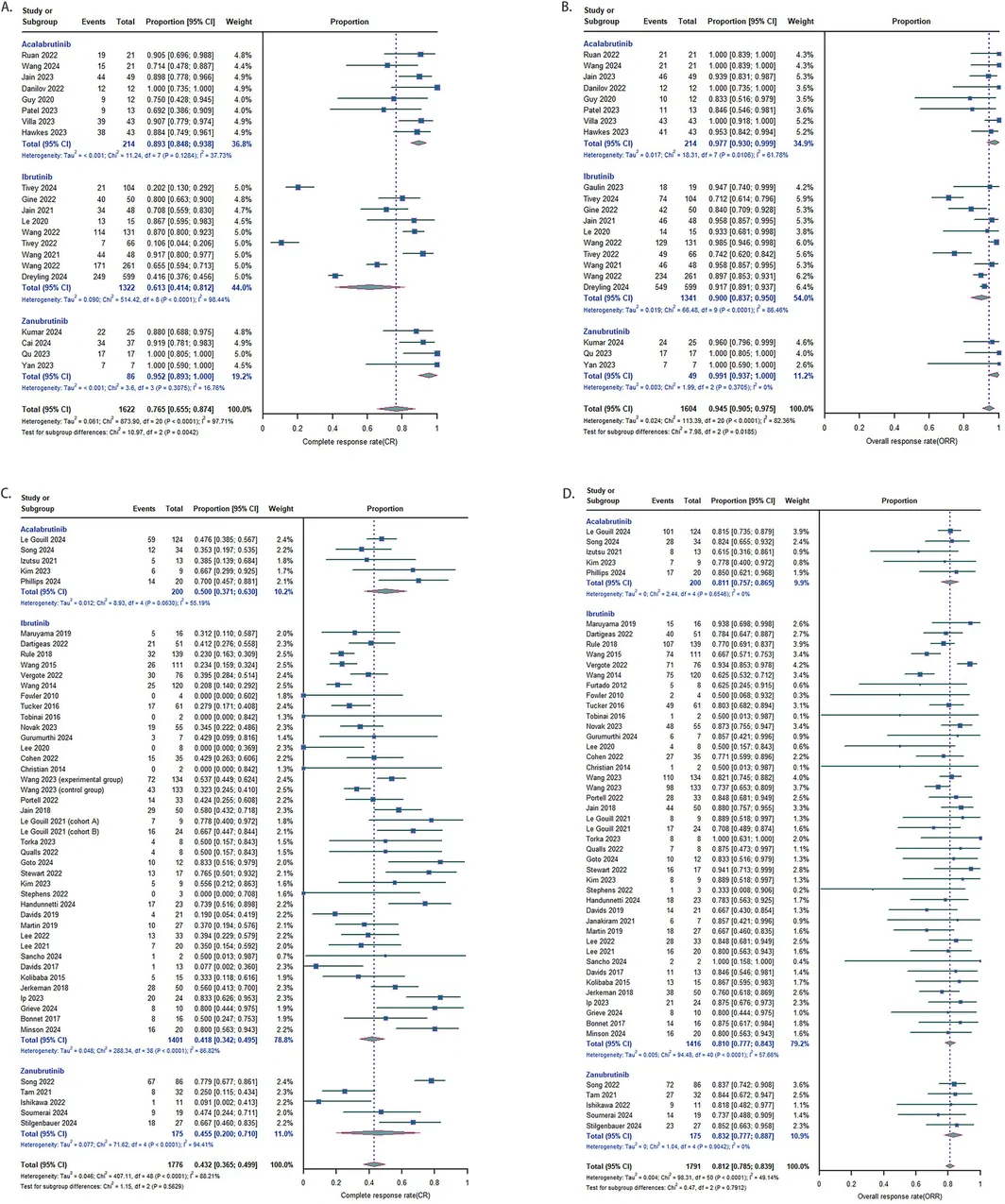
The effect per types of BTKi. (A) CR rate for treatment‐naïve patients; (B) ORR for treatment‐naïve patients; (C) CR rate for R/R patients; (D) ORR for R/R patients. BTKis, bruton tyrosine kinase inhibitors; CR, complete remission; ORR, objective response rate; R/R, relapsed/refractory.

Figure 3 showed the subgroup analyses stratified by types of BTKis (acalabrutinib, ibrutinib and zanubrutinib). Zanubrutinib therapy showed a significantly higher CR rate in the TN group compared with acalabrutinib and ibrutinib therapies (Zanubrutinib, 95.2% [95% CI 0.893, 1.000]; Acalabrutinib, 89.3% [95% CI 0.848, 0.938]; Ibrutinib, 61.3% [95% CI 0.414, 0.812]; *p* = 0.0042; Figure 3A). And the ORR of ibrutinib therapy for TN patients was lower than other two therapies (Ibrutinib, 90.0% [95% CI 0.837, 0.950]; Acalabrutinib, 97.7% [95% CI 0.930, 0.999]; Zanubrutinib, 99.1% [95% CI 0.937, 1.000]; *p* = 0.0185; Figure 3B). For R/R patients, these three BTKis have similar efficacy (CR rate, *p* = 0.5629; ORR, *p* = 0.7912; Figure 3C,D).

### 3.6 Subgroup Analysis: Response Rates by the Intervention Types

The intervention types included: BTKi monotherapy; BTKi + anti‐CD20 monoclonal antibody; BTKi + anti‐CD20 monoclonal antibody + traditional chemotherapy ± HSCT; BTKi + anti‐CD20 monoclonal antibody + small‐molecular therapy (proteasome inhibitors, venetoclax or lenalidomide); BTKi + small‐molecular therapy (proteasome inhibitors, venetoclax or lenalidomide); BTKi + other therapy which was not recommended in the NCCN guideline (version 2025); BTKi + anti‐CD20 monoclonal antibody + other therapy which was not recommended in the NCCN guideline (version 2025); BTKi + CAR‐T. The small‐molecular therapy group and the other therapy group do not fall under traditional chemotherapy but constitute a vital part of chemotherapy‐free regimens, differing solely in the varying levels of recommendation currently outlined in the NCCN 2025 guideline.

**FIGURE 4:**
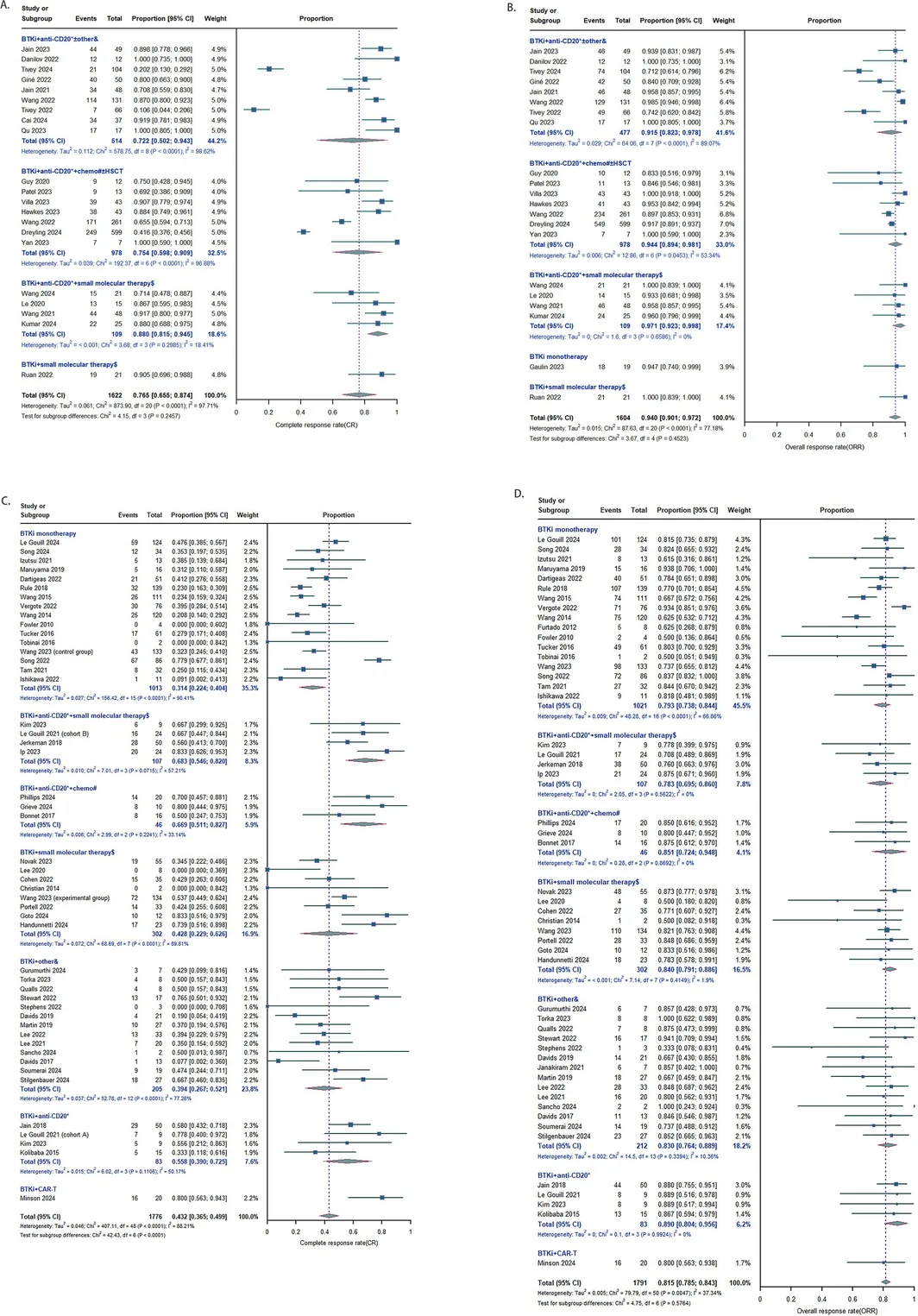
The effect by intervention types. (A) CR rate for treatment‐naïve patients. (B) ORR for treatment‐naïve patients; (C) CR rate for R/R patients; (D) ORR for R/R patients. *Anti‐CD20: Anti‐CD20 monoclonal antibodies, including rituximab, obinituzumab and ublituximab. #chemo: Traditional chemotherapies, including CHOP, DHAP, DHAOx, bendamustine and cytarabine. $small‐molecular therapy: Non‐traditional chemotherapies recommended in NCCN guideline (version 2025), including proteasome inhibitor (bortezomib, carfilzomib, ixazomib), venetoclax and lenalidomide; &other: Other therapies which were not recommended in the NCCN guideline (version 2025). BTKi, bruton tyrosine kinase inhibitor; CAR‐T, chimeric antigen receptor T‐cell immunotherapy; CR, complete remission; HSCT, haematopoietic stem cell transplantation; ORR, objective response rate; R/R, relapsed/refractory.

Figure 4 displayed the efficacy of BTKi monotherapy and BTKis combined with other therapies. In the TN group, the combination of BTKi + anti‐CD20 monoclonal antibody + small‐molecular therapy presented slightly higher CR rate and ORR than those of the combination of BTKi + anti‐CD20 monoclonal antibody + traditional chemotherapy±HSCT, but the differences were not significant (CR rate: 88.0% [95% CI 0.815, 0.945] vs. 75.4% [95% CI 0.598, 0.909], *p* = 0.2457; ORR: 97.1% [95% CI 0.923, 0.998] vs. 94.4% [95% CI 0.894, 0.981], *p* = 0.4523; Figure 4A,B). In the R/R group, the BTKi + CAR‐T group and the BTKi + anti‐CD20 monoclonal antibody + small‐molecular therapy group showed better CR rates of 80.0% [95% CI 0.563, 0.943] and 68.3% [95% CI 0.546, 0.820], respectively (*p* < 0.0001; Figure 4C). However, there was no significant difference in ORR among these intervention therapies (*p* = 0.5764, Figure 4D).

### 3.7 Subgroup Analysis: Response Rates Per BTKis Monotherapy

**FIGURE 5:**
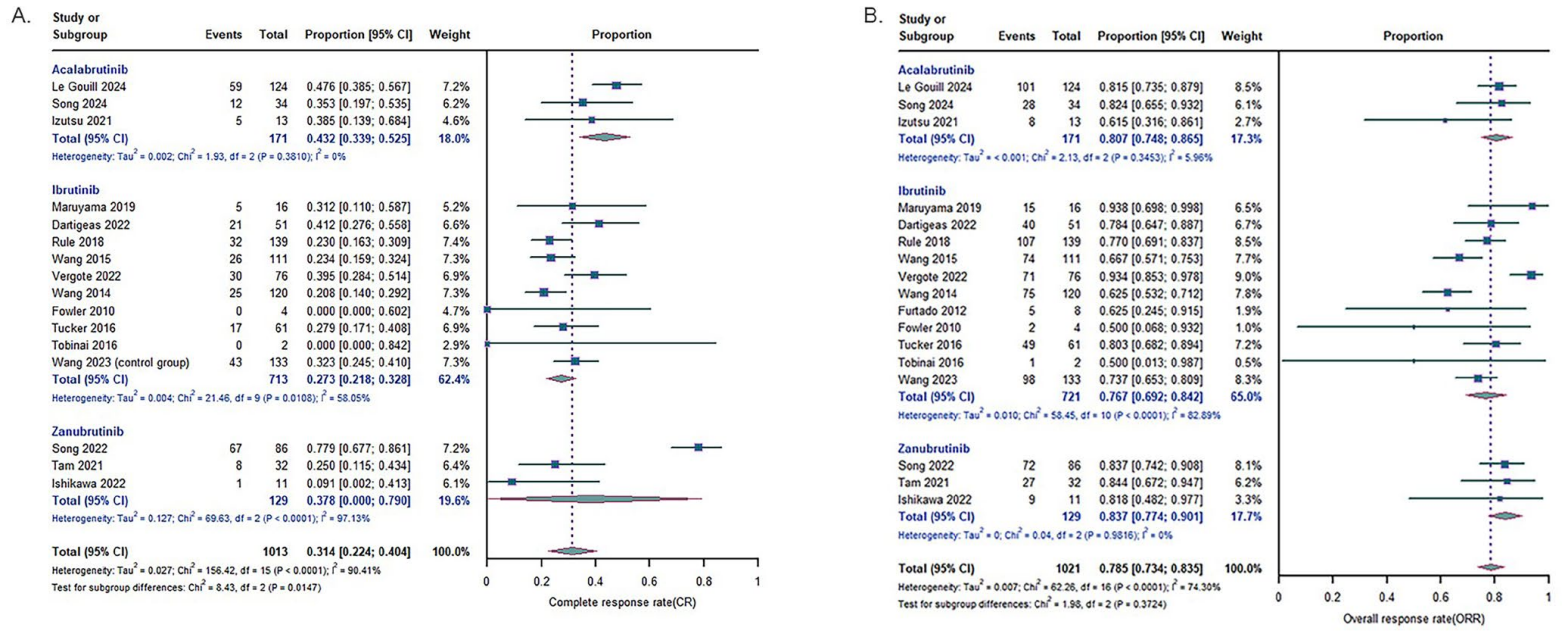
The efficacy of three types of BTKi monotherapy for R/R MCL patients. (A) CR rate; (B) ORR. BTKis, bruton tyrosine kinase inhibitors; CR, complete remission; ORR, objective response rate; R/R, relapsed/refractory.

There was only one study estimating the response rate of ibrutinib monotherapy in the TN group. Figure 5 presented the pooled analyses of the effectiveness of three BTKi monotherapies in the R/R group. Acalabrutinib monotherapy showed a higher CR rate than those of the other monotherapies (Acalabrutinib, 43.2% [95% CI 0.339, 0.525]; Ibrutinib, 27.3% [95% CI 0.218, 0.328]; Zanubrutinib, 37.8% [95% CI 0.000, 0.790]; *p* = 0.0147; Figure 5A). The ORR of these three BTKi monotherapies was similar (*p* = 0.3724, Figure 5B).

### 3.8 Toxicity

53 studies reported AE data. Neutropenia, thrombocytopenia, and anaemia were the main haematological AEs. In the TN group, the pooled rates for haematological AEs were 34.1% [95% CI 0.242, 0.440] for neutropenia, 33.2% [95% CI 0.173, 0.492] for thrombocytopenia, and 20.4% [95% CI 0.069, 0.339] for anaemia (Figure S2). In the R/R group, the pooled rates for haematological AEs were 28.2% [95% CI 0.200, 0.364] for neutropenia, 34.5% [95% CI 0.249, 0.440] for thrombocytopenia, and 16.6% [95% CI 0.124, 0.208] for anaemia (Figure S3). In addition, zanubrutinib‐based therapy exhibited a lower pooled rate of neutropenia for TN patients (48.8% [95% CI 0.328, 0.649] for acalabrutinib, 27.9% [95% CI 0.154, 0.403] for Ibrutinib, 20.0% [95% CI 0.023, 0.378] for zanubrutinib; *p* = 0.0411; Figure S4E) and a lower pooled rate of thrombocytopenia for R/R patients (33.7% [95% CI 0.184, 0.490] for acalabrutinib vs. 36.7% [95% CI 0.249, 0.484] for Ibrutinib vs. 13.7% [95% CI 0.017, 0.256] for zanubrutinib; *p* = 0.0175; Figure S5F) compared with the other two therapies.

Infection was a common non‐haematological AE (32.0% [95% CI 0.188, 0.452] in the TN group, 39.6% [95% CI 0.307, 0.485] in the R/R group; Figures S2D and S3D). Zanubrutinib‐based therapy exhibited a higher pooled rate of infection compared with the other two therapies in the R/R group (53.7% [95% CI 0.360, 0.715] for acalabrutinib, 33.8% [95% CI 0.239, 0.436] for ibrutinib, 66.1% [95% CI 0.576, 0.747] for zanubrutinib; *p* < 0.0001; Figure S5D). However, no significant difference was observed in the TN group (*p* = 0.6974, Figure S4D). In the R/R group, the pooled rate of haemorrhage with acalabrutinib‐based therapy was significantly lower than those of the other two therapies (9.3% [95% CI 0.000, 0.210] for acalabrutinib, 25.7% [95% CI 0.131, 0.383] for ibrutinib, 44.9% [95% CI 0.253, 0.646] for zanubrutinib; *p* = 0.0066; Figure S5C); and the pooled rate of cardiac events with ibrutinib‐based therapy was significantly higher than those of the other two therapies (2.0% [95% CI 0.000, 0.042] for acalabrutinib, 7.7% [95% CI 0.049, 0.104] for ibrutinib, 0.2% [95% CI 0.000, 0.017] for zanubrutinib; *p* < 0.0001; Figure S5B). Conversely, the pooled rates of other non‐haematological AEs were similar among the three BTKi‐based therapies in the TN group (Figure S4A–C).

### 3.9 Publication Bias

The publication bias of the CR rate and ORR in the TN group and the R/R group was analysed using funnel plots, the Egger's test, and the Begg's test. The funnel plots for CR in the TN group and for ORR in both groups were approximately symmetrical (Figure S1), and the results of the Egger's test and Begg's test demonstrated that there was no potential publication bias.

### 3.10 Sensitivity Analyses

Sensitivity analyses were conducted by sequentially eliminating each study from the pooled results, revealing that the combined findings were sufficiently robust (Figure S6).

## 4 Discussion

This systematic review and meta‐analysis compared responses among the approved BTKis, including acalabrutinib, ibrutinib, and Zanubrutinib, in patients with TN and R/R MCL. Current practical recommendations suggest the use of BTKi‐based therapies in patients with R/R MCL and younger patients with TN MCL [83]. In a large‐scale Phase II study of ibrutinib monotherapy for R/R MCL, the ORR was 68% with a CR rate of 21% [9]. A randomized Phase III trial comparing ibrutinib with temsirolimus showed a significant improvement in PFS for patients treated with ibrutinib compared with those treated with temsirolimus [84].

Since the introduction of ibrutinib, further developments have been made, with the approval of second‐ and next‐generation BTKis. The 2025 NCCN guideline prioritizes acalabrutinib‐based combinations for TN MCL (based on two studies [14, 83]). Zanubrutinib and acalabrutinib, which are new covalent BTKis, demonstrated comparable or improved efficacy relative to ibrutinib with superior safety in previous trials of patients with CLL and WM [85]. However, no head‐to‐head studies have reported comparisons among acalabrutinib, ibrutinib and zanubrutinib for MCL. The findings of this meta‐analysis provided insights into the comparative response rates associated with these three BTKis. In the TN group, the CR/OR rates of acalabrutinib and zanubrutinib were 89.3%/97.7% and 95.2%/99.1%, respectively, while ibrutinib showed a lower CR rate of 61.3% and a lower ORR of 90.0%, respectively. For patients with R/R MCL, the response rates of zanubrutinib and acalabrutinib were comparable to those of ibrutinib, and were associated with fewer haematological toxicities and cardiac events. Based on these results, acalabrutinib‐ and zanubrutinib‐based therapies have the potential to augment first‐line therapeutic approaches.

However, it is worth noting that BTKi monotherapy is difficult to achieve deep and durable CR, and some patients will still experience disease progression due to acquired resistance, particularly in patients with R/R MCL [86]. In an effort to improve the prognosis, various combination therapies have been recommended. Chemoimmunotherapy has been the standard first‐line treatment for MCL patients and has produced high response rates and longer PFS [87]. Rituximab, an anti‐CD20 monoclonal antibody, is an established component of frontline immunochemotherapy for patients with MCL [88]. A Phase 2/3 randomized trial compared the combination of ibrutinib and rituximab with standard immunochemotherapy for previously untreated MCL. The median PFS of ibrutinib‐rituximab was superior to that of standard immunotherapy, and the Grade 3 or above adverse events occurring in the ibrutinib‐rituximab group were fewer than those in the immunochemotherapy group [89]. The ORR of acalabrutinib plus bendamustine‐rituximab for untreated elderly MCL patients could reach 91.0%, and this combination regimen resulted in a clinically meaningful improvement in PFS [83]. In this meta‐analysis, the CR rate of treatment regimen including BTKi and anti‐CD20 monoclonal antibody was significantly higher than that of BTKi monotherapy in the R/R group. Overall, the results indicate that this combination significantly improves upon BTKi monotherapy. Small‐molecular therapies, including venetoclax, lenalidomide, and proteasome inhibitors, can also increase treatment efficacy by targeting complementary disease‐signalling pathways beyond those targeted by BTKis. An observational cohort study demonstrated that BTKi‐venetoclax regimen could overcome the unfavourable prognosis of TP53‐mutated MCL with improvements in survival [90]. According to the results of subgroup analysis in our meta‐analysis, the BTKis combined with small‐molecular therapy have shown higher response rates compared with BTKi monotherapy for R/R MCL patients. Moreover, compared with the addition of chemotherapy, adding small‐molecular therapy to the combination therapy of BTKi and an anti‐CD20 monoclonal antibody significantly increased the CR rate in R/R MCL patients (68.3% vs. 66.9%), and also slightly enhanced the therapeutic efficacy for untreated MCL patients (CR rate: 75.4% vs. 88.0%; ORR: 94.4% vs. 97.1%). These results suggest a potential clinical benefit of chemotherapy‐free combination regimens.

This article provided a holistic overview of the efficacy of three types of BTKi (acalabrutinib, ibrutinib and zanubrutinib). However, despite assessing their quality and employing appropriate statistical methods, there were still some limitations. Firstly, most studies were single‐arm studies rather than RCTs, leading to patient selection bias or other uncontrolled confounding factors that may reduce the generalizability and strength of the evidence. Secondly, the analyses showed moderate‐to‐high heterogeneity, which may be related to the differences in patient populations (e.g., demographic and clinical characteristics, presence of TP53 mutation, treatment line) and duration of follow‐up. The limited availability of detailed patient information may also affect the reliability of the meta‐analysis results. In addition, numerous studies omitted reports on the details of survival outcomes (PFS and OS). Due to the unavailability of sufficient data from a majority of the included studies, a meta‐analysis for PFS and OS was not feasible. This precludes definitive conclusions regarding the long‐term efficacy and survival benefits of the intervention. Further studies with complete and standardized reporting of these endpoints are warranted to validate our findings. While CAR‐T therapy (Brexucabtagene autoleucel, Lisocabtagene maraleucel) has been incorporated into the 2025 NCCN guideline, only 20 patients in a clinical trial that was included in this systematic review received BTKi + CAR‐T therapy. Therefore, caution should be exercised when interpreting BTKi + CAR‐T efficacy. To address these gaps and shortcomings, more large‐scale randomized controlled studies are required.

In conclusion, this meta‐analysis resolved critical uncertainties in BTKi selection for MCL, demonstrating that acalabrutinib and zanubrutinib may be more promising than ibrutinib as first‐line treatment options for MCL, owing to their superior efficacy and more favourable safety profile. Chemotherapy‐free combination regimens can partially overcome the traditionally unfavourable prognosis associated with R/R MCL. The observed heterogeneity and the absence of survival analysis results underscore the need for further studies to confirm these results. These results provide a roadmap for optimizing MCL therapy.

## Author Contributions

**Fangfei Xu:** writing – original draft, writing – review and editing, data curation, formal analysis, methodology, resources, software, visualization. **Yulin Yang:** data curation, formal analysis, software, visualization. **Wei Huang:** conceptualization, project administration, writing – original draft, writing – review and editing, supervision. **Xiuping Zou:** formal analysis, investigation, validation, writing – review and editing. **Kuangguo Zhou:** investigation, methodology, supervision.

## Funding

The authors have nothing to report.

## Conflicts of Interest

The authors declare no conflicts of interest.

## Supporting information


**Figure S1:** (A) Funnel plot of the CR rate for treatment‐naïve patients; (B) Funnel plot of the ORR for treatment‐naïve patients; (C) Funnel plot of the CR rate for R/R patients; *D. funnel* plot of the ORR for R/R patients. CR, complete remission; ORR, objective remission rate; R/R, relapsed/refractory.
**Figure S2:** The forest plot for the adverse effect of BTKis for treatment‐naïve patients. (A) Hepatotoxicity; (B) cardiac event; (C) haemorrhage; (D) infection; (E) neutropenia; (F) thrombocytopenia; (G). Anaemia. BTKis, bruton tyrosine kinase inhibitors.
**Figure S3:** The forest plot for the adverse effect of BTKis for R/R patients. A. Hepatotoxicity; B. Cardiac event; C. Haemorrhage; D. Infection; E. Neutropenia; F. Thrombocytopenia; G. Anaemia. BTKis, bruton tyrosine kinase inhibitors; R/R, relapsed/refractory.
**Figure S4:** The adverse effect per types of BTKi for treatment‐naïve patients. (A) Hepatotoxicity; (B) cardiac event; (C) haemorrhage; (D) infection; (E) neutropenia; (F) thrombocytopenia; (G) anaemia. BTKis, bruton tyrosine kinase inhibitors.
**Figure S5:** The adverse effect per types of BTKi for R/R patients. (A) Hepatotoxicity; (B) cardiac event; (C) haemorrhage; (D) infection; (E) neutropenia; (F) thrombocytopenia; (G) anaemia. BTKis, bruton tyrosine kinase inhibitors; R/R, relapsed/refractory.
**Figure S6:** The graph of sensitivity analysis. (A) The sensitivity analysis of the total CR rate for treatment‐naïve patients; (B) The sensitivity analysis of the total ORR for treatment‐naïve patients; (C) The sensitivity analysis of the total CR rate for R/R patients; (D) The sensitivity analysis of the total ORR for R/R patients. CR, complete response; ORR, objective remission rate; R/R, relapsed/refractory.
**Table S1:** Search strategy and results of PubMed.
**Table S2:** Search strategy and results of Cochrane.
**Table S3:** Search strategy and results of Embase.
**Table S4:** Risk of bias assessment for single arm studies using the methodological index for non‐randomized studies. (A) A clearly stated aim; (B) inclusion of consecutive patients; (C) prospective collection of data; (D) endpoints appropriate to the aim of the study; (E) unbiased assessment of the study endpoint; (F) follow‐up period appropriate to the aim of the study; (G) loss to follow up less than 5%; (H) prospective calculation of the study size.
**Table S5:** Risk of bias assessment for randomized controlled trials using the Cochrane Risk of Bias 2 tool.

## Data Availability

This meta‐analysis was based solely on previously published data and did not involve the collection of new data. All source data are publicly available in the referenced trial publications. Intermediate datasets generated during the meta‐analysis are available from the corresponding author upon request.
