# Supplementary material for: Comparative Efficacy of BTK Inhibitors in Treatment‐Naïve and Relapsed/Refractory Mantle Cell Lymphoma: A Systematic Review and Meta‐Analysis
Source: J Cell Mol Med. 2026 Sep 2;30(17):e71340. doi: 10.1111/jcmm.71340 (PMC13538458; doi:10.1111/jcmm.71340)
Supplement: Supplementary file 1 [file JCMM-30-e71340-s001.docx]

**Supplementary Materials**

**Contents**

**Figure S1. A. Funnel plot of the CR rate for treatment-naïve patients; B. Funnel plot of the ORR for treatment-naïve patients; C. Funnel plot of the CR rate for R/R patients; D. Funnel plot of the ORR for R/R patients.** CR, complete remission; ORR, objective remission rate; R/R, relapsed/refractory.

**Figure S2.** **The forest plot for the adverse effect of BTKis for treatment-naïve patients. A. Hepatotoxicity; B. Cardiac event; C. Hemorrhage; D. Infection; E. Neutropenia; F. Thrombocytopenia; G. Anemia.** BTKis, bruton tyrosine kinase inhibitors.

**Figure S3.** **The forest plot for the adverse effect of BTKis for R/R patients. A. Hepatotoxicity; B. Cardiac event; C. Hemorrhage; D. Infection; E. Neutropenia; F. Thrombocytopenia; G. Anemia.** BTKis, bruton tyrosine kinase inhibitors; R/R, relapsed/refractory.

**Figure S4.** **The adverse effect per types of BTKi for treatment-naïve patients. A. Hepatotoxicity; B. Cardiac event; C. Hemorrhage; D. Infection; E. Neutropenia; F. Thrombocytopenia; G. Anemia.** BTKis, bruton tyrosine kinase inhibitors.

**Figure S5.** **The adverse effect per types of BTKi for R/R patients. A. Hepatotoxicity; B. Cardiac event; C. Hemorrhage; D. Infection; E. Neutropenia; F. Thrombocytopenia; G. Anemia.** BTKis, bruton tyrosine kinase inhibitors; R/R, relapsed/refractory.

**Figure S6. The graph of sensitivity analysis. A. The sensitivity analysis of the total CR rate for treatment-naïve patients; B. The sensitivity analysis of the total ORR for treatment-naïve patients; C. The sensitivity analysis of the total CR rate for R/R patients; D. The sensitivity analysis of the total ORR for R/R patients.** CR, complete response; ORR, objective remission rate; R/R, relapsed/refractory.

**Table S1. Search strategy and results of PubMed.**

**Table S2. Search strategy and results of Cochrane.**

**Table S3. Search strategy and results of Embase.**

**Table S4. Risk of bias assessment for single arm studies using the methodological index for non-randomized studies.** Notes: A, A clearly stated aim; B, Inclusion of consecutive patients; C, Prospective collection of data; D, Endpoints appropriate to the aim of the study; E, Unbiased assessment of the study endpoint; F, Follow-up period appropriate to the aim of the study; G, Loss to follow up less than 5%; H, Prospective calculation of the study size.

**Table S5. Risk of bias assessment for randomized controlled trials using the Cochrane Risk of Bias 2 tool.**

**Figure S1.** **A. Funnel plot of the CR rate for treatment-naïve patients; B. Funnel plot of the ORR for treatment-naïve patients; C. Funnel plot of the CR rate for R/R patients; D. Funnel plot of the ORR for R/R patients.** CR, complete remission; ORR, objective remission rate; R/R, relapsed/refractory.

**Figure S2.** **The forest plot for the adverse effect of BTKis for treatment-naïve patients. A. Hepatotoxicity; B. Cardiac event; C. Hemorrhage; D. Infection; E. Neutropenia; F. Thrombocytopenia; G. Anemia.** BTKis, bruton tyrosine kinase inhibitors.

**Figure S3.** **The forest plot for the adverse effect of BTKis for R/R patients. A. Hepatotoxicity; B. Cardiac event; C. Hemorrhage; D. Infection; E. Neutropenia; F. Thrombocytopenia; G. Anemia.** BTKis, bruton tyrosine kinase inhibitors; R/R, relapsed/refractory.

**Figure S4.** **The adverse effect per types of BTKi for treatment-naïve patients. A. Hepatotoxicity; B. Cardiac event; C. Hemorrhage; D. Infection; E. Neutropenia; F. Thrombocytopenia; G. Anemia.** BTKis, bruton tyrosine kinase inhibitors.

**Figure S5.** **The adverse effect per types of BTKi for R/R patients. A. Hepatotoxicity; B. Cardiac event; C. Hemorrhage; D. Infection; E. Neutropenia; F. Thrombocytopenia; G. Anemia.** BTKis, bruton tyrosine kinase inhibitors; R/R, relapsed/refractory.

**Figure S6. The graph of sensitivity analysis. A. The sensitivity analysis of the total CR rate for treatment-naïve patients; B. The sensitivity analysis of the total ORR for treatment-naïve patients; C. The sensitivity analysis of the total CR rate for R/R patients; D. The sensitivity analysis of the total ORR for R/R patients.** CR, complete response; ORR, objective remission rate; R/R, relapsed/refractory.

**Table S1.** **Search strategy and results of PubMed**

| # | Searches |
| --- | --- |
| Acalabrutinib |  |
| 1 | (Calquence[Title/Abstract]) OR (ACP-196[Title/Abstract]) OR (acalabrutinib[Title/Abstract]) |
| 2 | ("Lymphoma, Mantle-Cell"[Mesh]) OR (Diffuse Lymphocytic Lymphoma, Poorly Differentiated[Title/Abstract]) OR (Diffuse Lymphocytic Lymphoma, Poorly-Differentiated[Title/Abstract]) OR (Lymphoma, Lymphocytic, Diffuse, Intermediate Differentiated[Title/Abstract]) OR (Centrocytic Small-Cell Lymphomas[Title/Abstract]) OR (Centrocytic Small-Cell Lymphoma[Title/Abstract]) OR (Mantle-Zone Lymphoma[Title/Abstract]) OR (Mantle-Zone Lymphomas[Title/Abstract]) OR (Mantle Cell Lymphoma[Title/Abstract]) OR (Mantle-Cell Lymphomas[Title/Abstract]) |
| 3 | #1 AND #2 (Result: 92) |
| Ibrutinib |  |
| 1 | (ibrutinib[Title/Abstract]) OR (PCI 32765[Title/Abstract]) OR (PCI-32765[Title/Abstract]) OR (PCI32765[Title/Abstract]) OR (Imbruvica [Title/Abstract]) OR (1-((3R)-3-(4-amino-3-(4-phenoxyphenyl)-1H-pyrazolo(3,4-d)pyrimidin-1-yl)piperidin-1- yl)prop-2-en-1-one [Title/Abstract]) |
| 2 | ("Lymphoma, Mantle-Cell"[Mesh]) OR (Diffuse Lymphocytic Lymphoma, Poorly Differentiated[Title/Abstract]) OR (Diffuse Lymphocytic Lymphoma, Poorly-Differentiated[Title/Abstract]) OR (Lymphoma, Lymphocytic, Diffuse, Intermediate Differentiated[Title/Abstract]) OR (Centrocytic Small-Cell Lymphomas[Title/Abstract]) OR (Centrocytic Small-Cell Lymphoma[Title/Abstract]) OR (Mantle-Zone Lymphoma[Title/Abstract]) OR (Mantle-Zone Lymphomas[Title/Abstract]) OR (Mantle Cell Lymphoma[Title/Abstract]) OR (Mantle-Cell Lymphomas[Title/Abstract]) |
| 3 | #1 AND #2 (Result: 573) |
| Zanubrutinib |  |
| 1 | (zanubrutinib[Title/Abstract]) OR (BGB-3111[Title/Abstract]) OR (Brukins[Title/Abstract]) OR ((7S)-2-(4-phenoxyphenyl)-7-(1-(prop-2-enoyl)piperidin-4-yl)-4,5,6,7-tetrahydropyrazolo(1,5-a)pyrimidine-3-carboxamide[Title/Abstract]) OR (7-(1-acryloyl-4-piperidinyl)-2-(4-phenoxyphenyl)-4,5,6,7-tetrahydropyrazolo(1,5-a)pyrimidine-3-carboxamide[Title/Abstract]) |
| 2 | ("Lymphoma, Mantle-Cell"[Mesh]) OR (Diffuse Lymphocytic Lymphoma, Poorly Differentiated[Title/Abstract]) OR (Diffuse Lymphocytic Lymphoma, Poorly-Differentiated[Title/Abstract]) OR (Lymphoma, Lymphocytic, Diffuse, Intermediate Differentiated[Title/Abstract]) OR (Centrocytic Small-Cell Lymphomas[Title/Abstract]) OR (Centrocytic Small-Cell Lymphoma[Title/Abstract]) OR (Mantle-Zone Lymphoma[Title/Abstract]) OR (Mantle-Zone Lymphomas[Title/Abstract]) OR (Mantle Cell Lymphoma[Title/Abstract]) OR (Mantle-Cell Lymphomas[Title/Abstract]) |
| 3 | #1 AND #2 (Result: 76) |

**Table S2.** **Search strategy and results of Cochrane**

| # | Searches |
| --- | --- |
| Acalabrutinib |  |
| 1 | (Calquence[Title/Abstract]) OR (ACP-196[Title/Abstract]) OR (acalabrutinib[Title/Abstract]) |
| 2 | ("Lymphoma, Mantle-Cell"[Mesh]) OR (Diffuse Lymphocytic Lymphoma, Poorly Differentiated[Title/Abstract]) OR (Diffuse Lymphocytic Lymphoma, Poorly-Differentiated[Title/Abstract]) OR (Lymphoma, Lymphocytic, Diffuse, Intermediate Differentiated[Title/Abstract]) OR (Centrocytic Small-Cell Lymphomas[Title/Abstract]) OR (Centrocytic Small-Cell Lymphoma[Title/Abstract]) OR (Mantle-Zone Lymphoma[Title/Abstract]) OR (Mantle-Zone Lymphomas[Title/Abstract]) OR (Mantle Cell Lymphoma[Title/Abstract]) OR (Mantle-Cell Lymphomas[Title/Abstract]) |
| 3 | #1 AND #2 (Result: 30) |
| Ibrutinib |  |
| 1 | (ibrutinib[Title/Abstract]) OR (PCI 32765[Title/Abstract]) OR (PCI-32765[Title/Abstract]) OR (PCI32765[Title/Abstract]) OR (Imbruvica [Title/Abstract]) OR (1-((3R)-3-(4-amino-3-(4-phenoxyphenyl)-1H-pyrazolo(3,4-d)pyrimidin-1-yl)piperidin-1- yl)prop-2-en-1-one [Title/Abstract]) |
| 2 | ("Lymphoma, Mantle-Cell"[Mesh]) OR (Diffuse Lymphocytic Lymphoma, Poorly Differentiated[Title/Abstract]) OR (Diffuse Lymphocytic Lymphoma, Poorly-Differentiated[Title/Abstract]) OR (Lymphoma, Lymphocytic, Diffuse, Intermediate Differentiated[Title/Abstract]) OR (Centrocytic Small-Cell Lymphomas[Title/Abstract]) OR (Centrocytic Small-Cell Lymphoma[Title/Abstract]) OR (Mantle-Zone Lymphoma[Title/Abstract]) OR (Mantle-Zone Lymphomas[Title/Abstract]) OR (Mantle Cell Lymphoma[Title/Abstract]) OR (Mantle-Cell Lymphomas[Title/Abstract]) |
| 3 | #1 AND #2 (Result: 118) |
| Zanubrutinib |  |
| 1 | (zanubrutinib[Title/Abstract]) OR (BGB-3111[Title/Abstract]) OR (Brukins[Title/Abstract]) OR ((7S)-2-(4-phenoxyphenyl)-7-(1-(prop-2-enoyl)piperidin-4-yl)-4,5,6,7-tetrahydropyrazolo(1,5-a)pyrimidine-3-carboxamide[Title/Abstract]) OR (7-(1-acryloyl-4-piperidinyl)-2-(4-phenoxyphenyl)-4,5,6,7-tetrahydropyrazolo(1,5-a)pyrimidine-3-carboxamide[Title/Abstract]) |
| 2 | ("Lymphoma, Mantle-Cell"[Mesh]) OR (Diffuse Lymphocytic Lymphoma, Poorly Differentiated[Title/Abstract]) OR (Diffuse Lymphocytic Lymphoma, Poorly-Differentiated[Title/Abstract]) OR (Lymphoma, Lymphocytic, Diffuse, Intermediate Differentiated[Title/Abstract]) OR (Centrocytic Small-Cell Lymphomas[Title/Abstract]) OR (Centrocytic Small-Cell Lymphoma[Title/Abstract]) OR (Mantle-Zone Lymphoma[Title/Abstract]) OR (Mantle-Zone Lymphomas[Title/Abstract]) OR (Mantle Cell Lymphoma[Title/Abstract]) OR (Mantle-Cell Lymphomas[Title/Abstract]) |
| 3 | #1 AND #2 (Result: 18) |

**Table S3.** **Search strategy and results of Embase.**

| # | Searches |
| --- | --- |
| Acalabrutinib |  |
| 1 | (('mantle cell lymphoma'/exp OR 'mantle cell lymphoma') AND [embase]/lim) |
| 2 | ((('lymphoma, mantle cell':ab,ti OR 'lymphoma, mantle-cell':ab,ti OR 'mantle cell lymphoma mcl':ab,ti OR 'mantle-cell lymphoma':ab,ti OR 'mantle-zone lymphoma':ab,ti OR mcl:ab,ti) AND 'mantle cell lymphoma':ab,ti OR 'mantle cell lymphoma':ab,ti) AND [embase]/lim) |
| 3 | #1 OR #2 |
| 4 | (('acalabrutinib'/exp OR 'acalabrutinib') AND [embase]/lim) |
| 5 | ((((((('4 [8 amino 3 [1':ab,ti AND '1 oxo 2 butyn 1 yl':ab,ti AND '2 pyrrolidinyl] imidazo [1, 5 a] pyrazin 1 yl] n':ab,ti AND '2 pyridinyl':ab,ti AND benzamide:ab,ti OR '4 [8 amino 3 [1':ab,ti) AND '1 oxo 2 butyn 1 yl':ab,ti AND '2 pyrrolidyl] imidazo [1, 5 a] pyrazin 1 yl] n':ab,ti AND '2 pyridyl':ab,ti AND benzamide:ab,ti OR '4 [8 amino 3 [1':ab,ti) AND '1 oxobut 2 yn 1 yl':ab,ti AND 'pyrrolidin 2 yl] imidazo [1, 5 a] pyrazin 1 yl] n':ab,ti AND 'pyridin 2 yl':ab,ti AND benzamide:ab,ti OR '4 [8 amino 3 [1':ab,ti) AND '2 butynoyl':ab,ti AND '2 pyrrolidinyl] imidazo [1, 5 a] pyrazin 1 yl] n':ab,ti AND '2 pyridinyl':ab,ti AND benzamide:ab,ti OR '4 [8 amino 3 [1':ab,ti) AND '2 butynoyl':ab,ti AND '2 pyrrolidyl] imidazo [1, 5 a] pyrazin 1 yl] n':ab,ti AND '2 pyridyl':ab,ti AND benzamide:ab,ti OR '4 [8 amino 3 [1':ab,ti) AND 'but 2 ynoyl':ab,ti AND 'pyrrolidin 2 yl] imidazo [1, 5 a] pyrazin 1 yl] n':ab,ti AND 'pyridin 2 yl':ab,ti AND benzamide:ab,ti OR 'acalabrutinib 2 butenedioate':ab,ti OR 'acalabrutinib maleate':ab,ti OR 'acp 196':ab,ti OR 'acp196':ab,ti OR 'calquence':ab,ti OR 'cml 1476':ab,ti OR 'cml1476':ab,ti OR 'org 3001960':ab,ti OR 'org3001960':ab,ti OR 'sch 900850':ab,ti OR 'sch900850':ab,ti OR 'acalabrutinib':ab,ti) AND [embase]/lim) |
| 6 | #4 OR #5 |
| 7 | #3 AND #6 (Result: 593) |
| Ibrutinib |  |
| 1 | 'ibrutinib'/exp |
| 2 | '1 [3 [4 amino 3':ab,ti AND '4 phenoxyphenyl':ab,ti AND '1h pyrazolo [3, 4 d] pyrimidin 1 yl] 1 piperidinyl] 2 propen 1 one':ab,ti OR '1 [3 [4 amino 3':ab,ti) AND '4 phenoxyphenyl':ab,ti AND '1h pyrazolo [3, 4 d] pyrimidin 1 yl] 1 piperidinyl] prop 2 en 1 one':ab,ti OR '1 [3 [4 amino 3':ab,ti) AND '4 phenoxyphenyl':ab,ti AND '1h pyrazolo [3, 4 d] pyrimidin 1 yl] piperidin 1 yl] prop 2 en 1 one':ab,ti OR 'cra 032765':ab,ti OR 'cra032765':ab,ti OR 'imbruvica':ab,ti OR 'jnj 54179060':ab,ti OR 'jnj54179060':ab,ti OR 'pci 32765':ab,ti OR 'pci 32765 00':ab,ti OR 'pci 32765-00':ab,ti OR 'pci32765':ab,ti OR 'pci32765 00':ab,ti OR 'pci32765-00':ab,ti OR 'ibrutinib':ab,ti |
| 3 | #1 OR #2 |
| 4 | 'mantle cell lymphoma'/exp |
| 5 | 'mantle cell lymphoma' |
| 6 | 'lymphoma, mantle cell':ab,ti OR 'lymphoma, mantle-cell':ab,ti OR 'mantle cell lymphoma mcl':ab,ti OR 'mantle-cell lymphoma':ab,ti OR 'mantle-zone lymphoma':ab,ti OR mcl:ab,ti) AND 'mantle cell lymphoma':ab,ti OR 'mantle cell lymphoma':ab,ti |
| 7 | #4 OR #5 OR #6 |
| 8 | #3 AND #7 (Result: 2707) |
| Zanubrutinib |  |
| 1 | 'zanubrutinib'/exp |
| 2 | 'zanubrutinib' |
| 3 | 2:ab,ti AND '4 phenoxyphenyl':ab,ti AND '7 [1':ab,ti AND '2 propenoyl':ab,ti AND '4 piperidinyl] 4, 5, 6, 7 tetrahydropyrazolo [1, 5 a] pyrimidine 3 carboxamide':ab,ti OR 2:ab,ti) AND '4 phenoxyphenyl':ab,ti AND '7 [1':ab,ti AND 'prop 2 enoyl':ab,ti AND 'piperidin 4 yl] 4, 5, 6, 7 tetrahydropyrazolo [1, 5 a] pyrimidine 3 carboxamide':ab,ti OR '4, 5, 6, 7 tetrahydro 7 [1':ab,ti) AND '1 oxo 2 propen 1 yl':ab,ti AND '4 piperidinyl] 2':ab,ti AND '4 phenoxyphenyl':ab,ti AND 'pyrazolo [1, 5 a] pyrimidine 3 carboxamide':ab,ti OR '4, 5, 6, 7 tetrahydro 7 [1':ab,ti) AND '1 oxo 2 propen 1 yl':ab,ti AND 'piperidin 4 yl] 2':ab,ti AND '4 phenoxyphenyl':ab,ti AND 'pyrazolo [1, 5 a] pyrimidine 3 carboxamide':ab,ti OR 'bgb 3111':ab,ti OR 'bgb3111':ab,ti OR 'brukinsa':ab,ti OR 'zanubrutinib':ab,ti |
| 4 | #1 OR #2 OR #3 |
| 5 | 'mantle cell lymphoma'/exp |
| 6 | 'mantle cell lymphoma' |
| 7 | 'lymphoma, mantle cell':ab,ti OR 'lymphoma, mantle-cell':ab,ti OR 'mantle cell lymphoma mcl':ab,ti OR 'mantle-cell lymphoma':ab,ti OR 'mantle-zone lymphoma':ab,ti OR mcl:ab,ti) AND 'mantle cell lymphoma':ab,ti OR 'mantle cell lymphoma':ab,ti |
| 8 | #5 OR #6 OR #7 |
| 9 | #4 AND #8 (Result: 392) |

**Table S4.** **Risk of bias assessment for single arm studies using the methodological index for non-randomized studies.** Notes: A, A clearly stated aim; B, Inclusion of consecutive patients; C, Prospective collection of data; D, Endpoints appropriate to the aim of the study; E, Unbiased assessment of the study endpoint; F, Follow-up period appropriate to the aim of the study; G, Loss to follow up less than 5%; H, Prospective calculation of the study size.

| First author(year) | Trial registration number | A | B | C | D | E | F | G | H | Total score |
| --- | --- | --- | --- | --- | --- | --- | --- | --- | --- | --- |
| Ruan 2022 | NA | 2 | 1 | 2 | 2 | 0 | 2 | 2 | 0 | 11 |
| Wang 2024 | NCT02717624 | 2 | 2 | 2 | 2 | 1 | 2 | 2 | 1 | 14 |
| Jain 2023 | NCT05214183 | 2 | 1 | 2 | 2 | 0 | 2 | 2 | 1 | 12 |
| Danilov 2022 | NCT04783415 | 2 | 2 | 2 | 2 | 0 | 1 | 2 | 1 | 12 |
| Guy 2020 | NCT03623373 | 2 | 2 | 2 | 2 | 0 | 2 | 2 | 1 | 13 |
| Patel 2023 | NCT04115631 | 2 | 2 | 2 | 2 | 0 | 2 | 2 | 1 | 13 |
| Villa 2023 | NCT04566887 | 2 | 2 | 2 | 2 | 0 | 2 | 2 | 1 | 13 |
| Hawkes 2023 | ACTRN12619000990123 | 2 | 1 | 2 | 1 | 0 | 2 | 2 | 0 | 10 |
| Gaulin 2023 | NCT03282396 | 2 | 2 | 1 | 2 | 0 | 2 | 2 | 1 | 12 |
| Tivey 2024 | NA | 2 | 2 | 2 | 2 | 0 | 2 | 2 | 0 | 12 |
| Giné 2022 | NCT02682641 | 2 | 2 | 2 | 2 | 0 | 2 | 2 | 1 | 13 |
| Jain 2021 | NCT01880567 | 2 | 2 | 2 | 2 | 0 | 2 | 2 | 1 | 13 |
| Le Gouill 2020 | NCT02558816 | 2 | 2 | 2 | 2 | 0 | 2 | 2 | 1 | 13 |
| Wang 2022 | NCT02427620 | 2 | 2 | 2 | 2 | 0 | 2 | 2 | 1 | 13 |
| Tivey 2022 | NA | 2 | 2 | 2 | 2 | 0 | 2 | 2 | 0 | 12 |
| Wang 2021 | NCT03710772 | 2 | 1 | 2 | 1 | 0 | 1 | 2 | 1 | 10 |
| Kumar 2024 | NCT03824483 | 2 | 2 | 2 | 2 | 0 | 2 | 2 | 1 | 13 |
| Cai 2024 | NCT04624958 | 2 | 1 | 2 | 1 | 0 | 2 | 2 | 0 | 10 |
| Qu 2023 | NCT05504603 | 2 | 2 | 2 | 1 | 0 | 2 | 2 | 0 | 11 |
| Yan 2023 | NCT04736914 | 2 | 2 | 2 | 2 | 0 | 2 | 2 | 0 | 12 |
| Le Gouill 2024 | NCT02213926 | 2 | 2 | 2 | 2 | 0 | 2 | 2 | 1 | 13 |
| Song 2024 | NCT03932331 | 2 | 2 | 2 | 2 | 2 | 2 | 2 | 1 | 15 |
| Izutsu 2021 | NCT03198650 | 2 | 2 | 2 | 2 | 0 | 2 | 2 | 1 | 13 |
| Kim 2023 | NCT04855695 | 2 | 2 | 2 | 2 | 0 | 2 | 2 | 1 | 13 |
| Phillips 2024 | NCT02717624 | 2 | 2 | 2 | 2 | 0 | 2 | 2 | 1 | 13 |
| Maruyama 2019 | NCT02169180 | 2 | 2 | 2 | 1 | 0 | 2 | 2 | 0 | 11 |
| Dartigeas 2022 | NCT03425591 | 2 | 1 | 2 | 1 | 0 | 2 | 2 | 1 | 11 |
| Wang 2015 | NCT01236391 | 2 | 2 | 2 | 2 | 0 | 2 | 2 | 1 | 13 |
| Vergote 2022 | NA | 2 | 2 | 2 | 1 | 0 | 2 | 2 | 0 | 11 |
| Wang 2014 | NA | 2 | 2 | 2 | 2 | 2 | 2 | 2 | 0 | 14 |
| Furtado 2012 | NA | 2 | 1 | 2 | 1 | 0 | 2 | 2 | 0 | 10 |
| Fowler 2010 | NA | 2 | 2 | 2 | 1 | 0 | 2 | 2 | 0 | 11 |
| Tucker 2016 | NA | 2 | 2 | 2 | 1 | 0 | 2 | 2 | 0 | 11 |
| Tobinai 2016 | NCT01704963 | 2 | 2 | 1 | 2 | 0 | 2 | 2 | 1 | 12 |
| Novak 2023 | NCT02356458 | 2 | 2 | 2 | 2 | 0 | 2 | 2 | 1 | 13 |
| Gurumurthi 2024 | NCT03939182 | 2 | 1 | 2 | 2 | 0 | 2 | 2 | 1 | 12 |
| Lee 2020 | NA | 2 | 2 | 2 | 2 | 0 | 2 | 2 | 0 | 12 |
| Cohen 2022 | NA | 2 | 2 | 2 | 1 | 0 | 2 | 2 | 0 | 11 |
| Christian 2014 | NA | 2 | 2 | 2 | 1 | 0 | 2 | 2 | 0 | 11 |
| Portell 2022 | NCT02419560 | 2 | 2 | 2 | 2 | 0 | 2 | 2 | 1 | 13 |
| Jain 2018 | NCT01880567 | 2 | 2 | 2 | 1 | 0 | 2 | 2 | 1 | 12 |
| Le Gouill 2021 | NCT02558816 | 2 | 2 | 2 | 2 | 0 | 2 | 2 | 1 | 13 |
| Torka 2023 | NCT03479268 | 2 | 2 | 2 | 1 | 0 | 2 | 2 | 1 | 12 |
| Qualls 2022 | NCT03877055 | 2 | 2 | 2 | 2 | 0 | 2 | 2 | 1 | 13 |
| Goto 2024 | NCT04477486 | 2 | 2 | 2 | 2 | 2 | 2 | 2 | 1 | 15 |
| Stewart 2022 | NCT02756247 | 2 | 2 | 2 | 2 | 0 | 2 | 2 | 1 | 13 |
| Kim 2023 | NCT02736617 | 2 | 2 | 2 | 2 | 0 | 2 | 2 | 1 | 13 |
| Stephens 2022 | NCT02303392 | 2 | 2 | 2 | 2 | 0 | 2 | 2 | 1 | 13 |
| Handunnetti 2024 | NCT02471391 | 2 | 2 | 2 | 2 | 0 | 2 | 2 | 1 | 13 |
| Davids 2019 | NCT02268851 | 2 | 2 | 2 | 2 | 0 | 2 | 2 | 1 | 13 |
| Janakiram 2021 | NCT03684694 | 2 | 2 | 1 | 1 | 0 | 2 | 2 | 1 | 11 |
| Martin 2019 | NCT02159755 | 2 | 2 | 2 | 2 | 0 | 2 | 2 | 1 | 13 |
| Lee 2022 | NCT03088878 | 2 | 2 | 2 | 1 | 0 | 2 | 2 | 1 | 12 |
| Lee 2021 | NA | 2 | 2 | 2 | 1 | 0 | 2 | 2 | 0 | 11 |
| Sancho 2024 | NCT03424122 | 2 | 2 | 2 | 1 | 0 | 2 | 2 | 1 | 12 |
| Davids 2017 | NA | 2 | 1 | 2 | 2 | 0 | 2 | 2 | 0 | 11 |
| Kolibaba 2015 | NA | 2 | 2 | 2 | 2 | 0 | 2 | 2 | 0 | 12 |
| Jerkeman 2018 | NCT02460276 | 2 | 2 | 2 | 2 | 0 | 2 | 2 | 0 | 12 |
| Ip 2023 | NCT02446236 | 2 | 1 | 2 | 2 | 0 | 2 | 2 | 1 | 12 |
| Grieve 2024 | NCT03295240 | 2 | 2 | 2 | 2 | 0 | 2 | 2 | 1 | 13 |
| Bonnet 2017 | NA | 2 | 1 | 2 | 2 | 0 | 2 | 2 | 0 | 11 |
| Minson 2024 | NCT04234061 | 2 | 2 | 2 | 2 | 0 | 2 | 2 | 0 | 12 |
| Song 2022 | NCT03206970 | 2 | 2 | 2 | 2 | 0 | 2 | 2 | 1 | 13 |
| Tam 2021 | NCT02343120 | 2 | 2 | 2 | 2 | 2 | 2 | 2 | 1 | 15 |
| Ishikawa 2022 | NCT04172246 | 2 | 2 | 2 | 2 | 0 | 2 | 2 | 1 | 13 |
| Soumerai 2024 | NCT02914938 | 2 | 2 | 2 | 2 | 0 | 2 | 2 | 1 | 13 |
| Stilgenbauer 2024 | NCT04277637 | 2 | 1 | 2 | 2 | 0 | 2 | 2 | 1 | 12 |

**Table S5.** **Risk of bias assessment for randomized controlled trials using the Cochrane Risk of Bias 2 tool.**

| First author(year);  (Trial registration number) | Bias arising from the randomization process | Bias due to deviations from intended interventions | Bias due to missing outcome data | Bias in measurement of the outcome | Bias in selection of the reported result | Overall quality |
| --- | --- | --- | --- | --- | --- | --- |
| Dreyling 2024  (NCT02858258) | Low | High | Low | Low | Low | Some concerns |
| Wang 2022  (NCT01776840) | Low | Low | Low | Low | Low | Low |
| Wang 2023  (NCT03112174) | Some concerns | Low | Low | Low | Low | Low |
| Rule 2018  (NCT01646021) | Some concerns | Low | Low | Low | Low | Some concerns |
